# Self-reported changes in alcohol and tobacco use during COVID-19: findings from the eastern part of WHO European Region

**DOI:** 10.1093/eurpub/ckac011

**Published:** 2022-02-07

**Authors:** Carolin Kilian, Maria Neufeld, Jakob Manthey, Sophiko Alavidze, Anastacia Bobrova, Orna Baron-Epel, Merita Berisha, Rabia Bilici, Kairat Davletov, Laura Isajeva, Fatma Kantaş Yılmaz, Tatsiana Karatkevich, Alibek Mereke, Sanja Musić Milanović, Kristine Galstyan, Ljiljana Muslić, Michail Okoliyski, Zana Shabani, Mindaugas Štelemėkas, Lela Sturua, Sharon R Sznitman, Başak Ünübol, Carina Ferreira-Borges, Jürgen Rehm

**Affiliations:** Institute of Clinical Psychology and Psychotherapy, Technische Universität Dresden, Dresden, Germany; Institute of Clinical Psychology and Psychotherapy, Technische Universität Dresden, Dresden, Germany; World Health Organization European Office for Prevention and Control of Noncommunicable Diseases, Moscow, Russian Federation; Institute for Mental Health Policy Research, Centre for Addiction and Mental Health, Toronto, Ontario, Canada; Institute of Clinical Psychology and Psychotherapy, Technische Universität Dresden, Dresden, Germany; Centre for Interdisciplinary Addiction Research, University Medical Care Hamburg-Eppendorf, Hamburg, Germany; Department of Psychiatry, Medical Faculty, University of Leipzig, Leipzig, Germany; National Center for Disease Control and Public Health, Tbilisi, Georgia; National Academy of Sciences of Belarus Institute of Economics, Minsk, Belarus; School of Public Health, University of Haifa, Haifa, Israel; Department for Social Medicine, National Institute of Public Health of Kosova, Pristina, Kosovo; Cathedra for Social Medicine, Medical Faculty, University of Prishtina, Pristina, Kosovo; Erenköy Mental Health and Neurological Diseases Training and Research Hospital, University of Health Sciences Turkey, Istanbul, Turkey; Al-Farabi Kazakh National University, Almaty, Kazakhstan; Centre for Disease Prevention and Control, Riga, Latvia; Department of Health Management, University of Health Sciences Turkey, Istanbul, Turkey; Republican Scientific and Practical Centre for Mental Health, Minsk, Belarus; Al-Farabi Kazakh National University, Almaty, Kazakhstan; Croatian Institute of Public Health, Zagreb, Croatia; School of Medicine, University of Zagreb, Zagreb, Croatia; Public Health Department, Ministry of Health of the Republic of Armenia, Yerevan, Armenia; Croatian Institute of Public Health, Zagreb, Croatia; WHO Country Office in Bulgaria, Sofia, Bulgaria; Ministry of Health in Kosovo, Pristina, Kosovo; Health Research Institute, Faculty of Public Health, Lithuanian University of Health Sciences, Kaunas, Lithuania; Department of Preventive Medicine, Faculty of Public Health, Lithuanian University of Health Sciences, Kaunas, Lithuania; National Center for Disease Control and Public Health, Tbilisi, Georgia; Petre Shotadze Tbilisi Medical Academy, Tbilisi, Georgia; School of Public Health, University of Haifa, Haifa, Israel; Erenköy Mental Health and Neurological Diseases Training and Research Hospital, University of Health Sciences Turkey, Istanbul, Turkey; World Health Organization European Office for Prevention and Control of Noncommunicable Diseases, Moscow, Russian Federation; Institute of Clinical Psychology and Psychotherapy, Technische Universität Dresden, Dresden, Germany; Institute for Mental Health Policy Research, Centre for Addiction and Mental Health, Toronto, Ontario, Canada; Centre for Interdisciplinary Addiction Research, University Medical Care Hamburg-Eppendorf, Hamburg, Germany; Dalla Lana School of Public Health, University of Toronto, Toronto, Ontario, Canada; Faculty of Medicine, Institute of Medical Science, University of Toronto, Toronto, Ontario, Canada; Centre for Addiction and Mental Health, Campbell Family Mental Health Research Institute, Toronto, Ontario, Canada; Department of Psychiatry, University of Toronto, Toronto, Ontario, Canada; I.M. Sechenov First Moscow State Medical University (Sechenov University), Moscow, Russian Federation

## Abstract

**Background:**

The COVID-19 pandemic might impact substance use behaviours around the globe. In this study, we investigate changes in alcohol and tobacco use in the second half of 2020 in countries of the eastern part of the WHO European Region.

**Methods:**

Self-reported changes in alcohol and tobacco use among 11 295 adults from 18 countries in the eastern part of the WHO European Region were collected between August 2020 and January 2021. The non-probabilistic sample was weighted for age, gender and education. For each country, proportions of respondents reporting a decrease, no change or increase in substance use over the past 3 months were examined, and multinomial regression models were used to test associations with age, gender and past-year alcohol use.

**Results:**

In most countries, about half of the respondents indicating past-year alcohol or tobacco use reported no change in their substance use. Of those alcohol users who reported changes in their alcohol use, a larger proportion reported a decrease than an increase in most countries. The opposite was true for tobacco use. Women, young adults and past-year harmful alcohol users were identified as being more likely to change their substance use behaviour.

**Conclusion:**

We found diverging overall trends for alcohol and tobacco use in the second half of 2020. The patterns of change vary according to age, gender and past-year substance use. Individuals at risk to increase their substance use during the COVID-19 pandemic require most policy considerations.

## Introduction

Alcohol and tobacco use constitutes major risk factors for morbidity and mortality in the WHO European Region.^1^ In Eastern more so than in other European countries, substantial declines in the use of both substances and the attributable disease burden have been recorded in the last decade,^2^ and, at least for alcohol, this part of the Region is the main driving force behind the entire Region being on track to reach the Global NCD targets.^3^

As hypothesized in early 2020,^4^^,^^5^ the global pandemic of the coronavirus disease and the measures undertaken to contain the spread of the virus, such as physical distancing and closing of typical drinking locations such as bars and restaurants as part of the lockdown procedures (for an overview, see ^6^), appear to have a sizeable impact on substance use behaviour. The literature on the pandemic-driven variations in substance use not only in Europe, but also worldwide, is constantly growing, yet not conclusive in every aspect, and some key themes do solidify. First, preliminary sales data of single countries point at a reduction of alcohol use and to an increased use of tobacco, in 2020,^7–9^ which is also corroborated in a survey of 36 000 substance users from 21 Western and Central European countries.^10^ Second, changes in substance use are not uniform but do vary with age, gender and prior substance use. With regard to gender, some surveys report women to be less likely to increase their alcohol use,^11^ while most surveys converge on a higher likelihood for women to increase alcohol and/or tobacco use.^12–14^ The findings on variations with age groups are also not quite consistent. In two independent cross-sectional surveys of German adults, the lowest rates of increasing alcohol use were found in 55- to 64-year olds^15^ and in 15- to 34-year olds.^16^ In a French survey, self-reported increases in tobacco and alcohol use were highest among 16- to 29- and 30- to 49-year olds.^13^ Survey data from the UK suggest that older adults were less likely to both drink less and drink more during the pandemic.^12^ While findings on gender and age-related variations in legal substance use in Europe do differ across studies, the link to prior substance use is clearer. In the survey conducted in 21 Central and Western European countries, a continuous dose–response relationship could be identified between alcohol intake before the pandemic and changes in alcohol use during the pandemic,^15^^,^^17^^,^^18^ suggesting high probabilities to increase drinking among harmful alcohol users.

Against this backdrop, it is very likely that the COVID-19 pandemic impacts on the downward trend of alcohol and tobacco use in the eastern part of the WHO European Region. However, there is little evidence on changes in substance use during the pandemic in this part of the Region. In this report, we aim to explore how alcohol and tobacco use have changed in the second half of 2020 in the general adult population of countries of the eastern part of the WHO European Region. Based on the available literature on changes in drinking patterns during the pandemic, we expected alcohol use levels to decline on average and tobacco use levels to increase on average. Additionally, we study associations of substance use changes with gender, age groups and past-year substance use. While the literature does not allow us to draw specific conclusions on variations by gender and age group, we hypothesized that prior harmful alcohol use is significantly linked to changes in alcohol use.

## Methods

The study employed a cross-sectional survey to capture self-reported changes in alcohol and tobacco use following the COVID-19 pandemic. The target population included adults 18 years or older from the general population of countries of the eastern part of the WHO European Region (for a complete list of countries, see table 1). The survey was developed in English and translated into national and official languages by native speakers being study partners of the project. Altogether, the survey was available in more than 20 languages and disseminated across 23 countries between August 2020 and January 2021. To reach out the target population, the survey was made available via our study website (www.covid19-and-alcohol.eu) and was communicated mainly through social media and institutional web pages (non-probabilistic, convenience sampling). Participation to the survey was fully anonymous. The final set of countries included was those that had at least 300 observations (for sample size calculations, see ^19^).

**Table 1:** Sample characteristics by country, *n *=* *11 295

Country	Sample size	Gender, % women^a^	Age			Educational attainment: high school or lower	Past-year alcohol users	Past-year tobacco users^b^
			18–34 years	35–54 years	55+ years			
Armenia	366	54.2 (43.5–64.6)	21.6 (14.0–32.0)	64.3 (53.6–73.7)	14.1 (8.2–23.1)	35.8 (25.0–48.2)	86.8 (78.6–92.1)	35.9 (25.7–47.7)
Belarus	516	53.3 (45.6–60.9)	38.3 (31.0–46.0)	32.6 (26.3–39.7)	29.1 (22.5–36.7)	46.2 (38.3–54.3)	90.4 (86.0–93.5)	28.1 (21.3–36.0)
Bulgaria	600	50.7 (43.6–57.9)	25.6 (20.3–31.7)	60.4 (53.4–66.9)	14.0 (9.9–19.4)	50.0 (42.9–57.2)	90.4 (84.5–94.2)	44.8 (37.7–52.2)
Croatia	554	50.8 (43.3–58.2)	27.6 (22.5–33.2)	57.2 (50.0–64.1)	15.2 (10.4–21.7)	77.7 (73.4–81.5)	79.3 (71.9–85.2)	46.2 (38.8–53.8)
Estonia	347	51.5 (41.7–61.1)	29.2 (21.8–37.7)	53.5 (44.0–62.8)	17.3 (11.7–24.9)	62.2 (53.9–69.7)	87.0 (78.1–92.7)	39.4 (30.0–49.7)
Georgia	604	51.4 (43.6–59.1)	48.3 (40.5–56.1)	39.1 (32.0–46.8)	12.6 (9.2–17.1)	48.6 (40.8–56.5)	74.5 (66.8–80.9)	48.0 (40.1–55.9)
Israel	454	53.6 (47.4–59.6)	32.4 (27.6–37.7)	39.5 (33.8–45.6)	28.0 (22.2–34.6)	59.4 (54.0–64.6)	80.4 (74.9–85.0)	36.4 (30.8–42.5)
Kazakhstan	457	55.9 (47.4–64.1)	64.0 (55.3–71.9)	27.4 (20.7–35.3)	8.7 (4.5–16.0)	42.8 (33.3–52.9)	60.1 (50.7–68.8)	22.8 (16.7–30.4)
Kosovo^c^	499	76.5 (70.8–81.4)	94.5 (91.5–96.5)	4.5 (2.7–7.4)	1.0 (0.4–2.6)	96.1 (95.2–96.8)	24.6 (19.6–30.4)	20.7 (16.0–26.4)
Kyrgyzstan	471	51.3 (41.7–60.9)	45.3 (36.4–54.6)	41.3 (31.7–51.6)	13.3 (8.5–20.3)	69.5 (57.8–79.1)	49.1 (39.7–58.7)	30.6 (21.0–42.2)
Latvia	1988	52.9 (49.1–56.7)	43.6 (40.0–47.4)	41.1 (37.4–45.0)	15.2 (12.7–18.1)	68.1 (65.3–70.7)	88.7 (85.9–91.0)	45.9 (42.0–49.8)
Lithuania	577	51.7 (42.5–60.8)	40.7 (32.2–49.7)	42.2 (33.3–51.6)	17.1 (11.3–25.2)	63.1 (55.6–69.9)	77.2 (67.4–84.7)	32.3 (24.0–42.0)
Moldova	660	53.8 (45.5–61.9)	21.5 (16.4–27.6)	59.9 (52.1–67.2)	18.7 (13.3–25.5)	75.0 (69.8–79.7)	73.7 (65.6–80.4)	23.6 (17.0–31.9)
Montenegro	471	50.5 (40.3–60.7)	53.6 (43.1–63.8)	37.1 (27.2–48.3)	9.3 (4.8–17.2)	77.4 (71.7–82.2)	79.9 (69.4–87.5)	48.0 (37.7–58.5)
Romania	1079	50.4 (44.3–56.4)	69.0 (63.4–74.0)	27.1 (22.3–32.5)	4.0 (2.4–6.5)	84.5 (82.1–86.7)	93.8 (90.1–96.2)	54.2 (48.1–60.3)
Russia	576	55.1 (48.6–61.5)	33.9 (28.8–39.4)	47.0 (40.7–53.5)	19.1 (13.6–26.1)	61.4 (55.9–66.5)	79.9 (73.6–84.9)	43.7 (37.3–50.3)
Turkey	466	49.6 (41.4–57.9)	36.4 (29.8–43.6)	49.9 (41.7–58.2)	13.7 (7.6–23.3)	57.3 (49.6–64.7)	45.4 (37.3–53.8)	33.3 (25.6–42.0)
Ukraine	610	54.0 (44.6–63.1)	35.6 (26.8–45.6)	54.4 (44.9–63.5)	10.0 (7.2–13.9)	63.8 (56.1–70.8)	86.7 (78.5–92.0)	29.4 (21.4–39.0)

a Proportion of those reporting other gender was <1% in all countries.

b *n *=* *10 916, <5% missings. All proportions were weighted according to gender, age, and educational attainment. 95% CIs are presented in brackets.

c All references to Kosovo should be understood within the framework of the UN Security Council Resolution 1244 (1999).

### Assessment of changes in alcohol and tobacco use

Respondents were asked whether they experienced changes in the frequency of drinking, quantity of alcohol consumed and frequency of heavy episodic drinking (HED) during the past 3 months. Five answer options were given, namely to drink ‘much less’, ‘slightly less’, ‘no change’, ‘slightly more’ or ‘much more’ plus an option to refuse responding.

With regard to tobacco use, a single variable asked whether the respondent had experienced changes in tobacco use over the past 3 months, with the same answer options as for alcohol, plus an additional option that tobacco is usually not used.

### Further variables of interest

Gender (women, men, other gender), age, educational attainment (less than high school, high school, any education beyond high school), and past-year alcohol use were recorded. The latter was measured using the three-item version of the Alcohol Use Disorder Identification Test AUDIT-C^20^ and a sum-score higher than eight indicated past-year harmful drinking.

For respondents with incomplete data due to missing information on either quantity of alcohol consumed or frequency of HED, ordinal logistic imputation was used to generate missing information by country based on the following variables: frequency of drinking, gender, age group and educational attainment. Respondents for whom responses were imputed did not substantially differ from respondents with complete data with regard to gender, age group and educational attainment (see Supplementary table S2). Across all countries, except for Belarus, 196 observations for drinking quantity and 143 observations for frequency of HED were imputed (<5%). In Belarus, only information on drinking frequency but not on the past-year quantity of pure alcohol consumed per drink day and the past-year frequency of HED was available, so the country was excluded in analysis taking into account harmful alcohol use.

### Statistical analysis

Because our sample represents a convenience sample, we calculated weights to adjust the sample distribution to the respective population distribution of each country according to gender, age group and educational attainment. We applied a multi-stage procedure in which gender, age and education strata were collapsed in ad hoc defined order to ensure that the resulting weights do not exceed a maximum weight of 10 (for details, see Supplement S1).

To statistically test average changes in alcohol use, we conducted weighted linear regression analyses. Given the high heterogeneity in the use of alcohol and tobacco across countries studied, results are presented by country only. Outcome variables were changes in (1a) frequency of drinking alcohol, (1b) quantity of alcohol consumed per occasion, (1c) frequency of HED and (2) frequency of tobacco use. Each indicator was rescaled by dividing the original values (−2 ‘much less’ to +2 ‘much more’) by two so that the resulting indicator had five levels between −1 to +1. We interpreted the significance and direction of the y-axis intercept, with negative values indicating an average decrease and positive values indicating an average increase in use. The regression models were weighted, yet not adjusted for any other variables.

Multivariate multinomial regression models were used to identify factors associated with decreases or increases in substance use. Models were run separately for (i) alcohol and (ii) tobacco. With regard to changes in alcohol use, all three change indicators were summed to a single indicator and rescaled again so that this indicator took values between −1 and +1. This approach assumes that changes in the different indicators can balance each other out. In other words, for those who indicated a substantial decrease or increase in all three indicators, the change score is −1 or +1, respectively, while for all others, the change score takes on values between −1 to 0 and 0 to +1, representing a combination of slight and substantial decreases and increases in change, respectively. Both outcome variables, i.e. the combined indicator for alcohol and the single indicator for tobacco use, were categorized with three groups: decrease (change score: <0), no change (change score: 0) and increase (change score: >0). The following independent variables were included in the models: gender (women, men), age group (18–34, 35–54, 55+ years) and harmful drinking (AUDIT-C sum-score ≥8). Respondents indicating themselves as of other gender were excluded in regression analyses due to their very low frequency (*n *=* *18, <1%). Multinomial regressions were adjusted for the fixed effects of country and the week in which a respondent took part in the survey. All analyses were done using Stata 15.1^21^ and R version 4.0.2.^22^

The study materials, data and codebooks are available at Figshare {*reference will be provided after acceptance for publication*} and may be used for further research.

## Results

**Figure 1:**
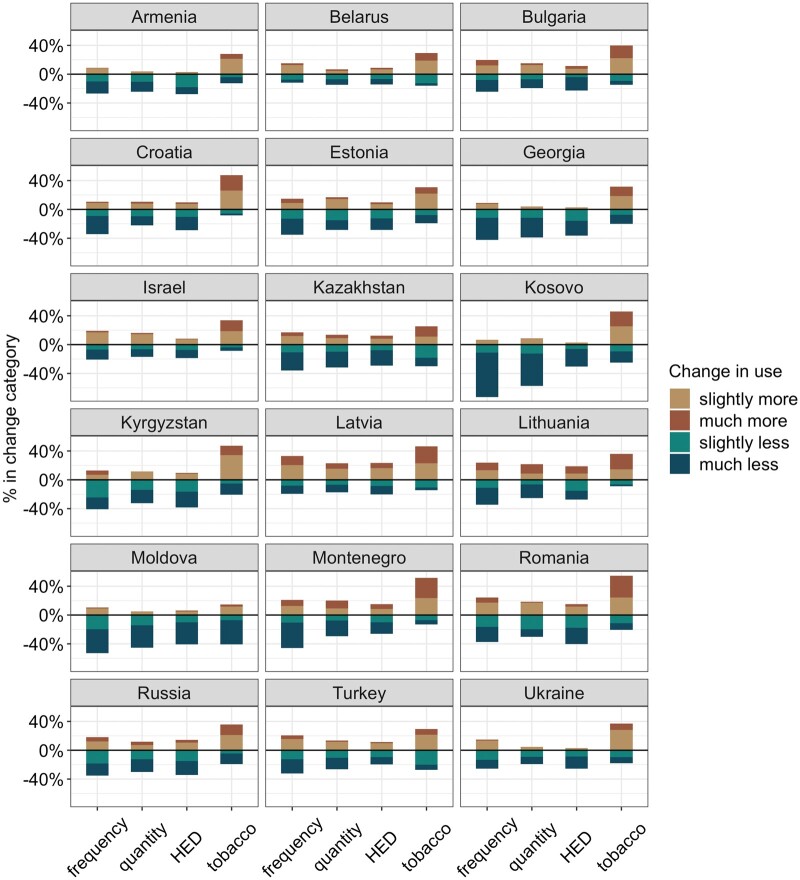
Proportion of respondents who report drinking and/or using tobacco much less, slightly less, slightly more and much more during the past three months, by country. Presented are changes in the drinking frequency (*n *=* *8831, <5% missing), the quantity of alcohol consumed per drink day (*n *=* *8815, <5% missing), the frequency in HED (*n *=* *8654, 5.4% missing) and the frequency of tobacco use (*n *=* *3688).

In total, 13 145 respondents took part in our survey, of which five countries were excluded since their sample sizes were too low (Azerbaijan, Bosnia and Herzegovina, North Macedonia, Serbia, and Uzbekistan), and <5% were excluded due to missing values in educational attainment (*n *=* *536, 4.1%) and drinking frequency (*n *=* *16, <0.01%). The final analytic sample included 11 295 adults from 18 countries. The sample size varied between 347 in Estonia and 1988 in Latvia. Women, younger respondents, and those with higher educational attainment were over-represented in all countries, with the gender, age and education distribution of the sample approximated to the actual population distribution after weighting (for a comparison of the unweighted data with the actual population distribution, see Supplementary table S1). Descriptive statistics are provided in table 1. Changes in substance use are illustrated in figure 1 (see also Supplementary tables S3 and S4 for sensitivity analysis including respondents with complete data on past-year alcohol use only).

### Changes in alcohol use

Prevalence of past-year alcohol users ranged between 24.6% [95% confidence interval (CI): 19.6–30.4] in Kosovo. (All references to Kosovo should be understood within the framework of the UN Security Council Resolution 1244 (1999).) to more than 90.0% in Bulgaria and Romania (see table 1). Among alcohol users, about half of the respondents reported that their frequency of drinking did not change, with the lowest proportion being observed in Kosovo (20.9%, 95% CI: 12.4–33.0) and the highest in Belarus (73.3%, 95% CI: 65.8–79.7), Armenia (64.5%, 95% CI: 52.4–75.0) and Ukraine (60.0%, 95% CI: 49.9–69.3). Significant negative change scores indicated that in most countries more respondents reported to decrease than to increase their drinking frequency. No substantial mean change in drinking frequency was observed in Belarus, Bulgaria, Israel, Kyrgyzstan, Lithuania and Turkey, while Latvia was the only country where the drinking frequency on average increased (*P* = 0.002, see Supplementary table S2).

With regard to changes in the drinking quantity and frequency of HED events, a similar picture emerged: at least half of the countries’ respondents reporting past-year alcohol use indicated no change, while among the remainders, on average, more respondents reported a decrease than an increase in their alcohol use, indicated by negative mean change scores. Decreases and increases in drinking quantity were on average about the same in Bulgaria, Israel, Latvia, Lithuania and Montenegro; for HED frequency this was the case in Latvia, Lithuania, Montenegro and Turkey (see Supplementary table S2). None of the countries reported average increases in the drinking quantity and HED frequency.

Men compared with women and young adults compared with middle-aged adults were more likely to report a decrease in their overall alcohol use compared with no change (table 2). In contrast, no gender effect was observed for an increase vs. no change in alcohol use, while young adults were again at higher risk to increase their drinking than middle-aged adults. Adults aged 55 years or over were, however, at lower risk for reporting an increase in drinking vs. no change compared with middle-aged adults. Respondents reporting past-year harmful drinking had four times the risk of increasing their alcohol use compared with no changes, while they were less likely to reduce their drinking compared with respondents with an AUDIT-C sum-score below the past-year harmful drinking threshold.

**Table 2:** Multinomial regression of self-reported decrease or increase in overall alcohol use compared with no change in overall alcohol use among past-year alcohol users, *n* = 8178

	Decrease vs. no change			Increase vs. no change		
	RRR	*P*	95% CI	RRR	*P*	95% CI
Gender: women (ref.: men)	0.81	<0.001	(0.72–0.90)	0.98	0.771	(0.86–1.12)
Age group (ref.: 35–54 years)						
18–34 years	1.47	<0.001	(1.32–1.63)	1.62	<0.001	(1.43–1.84)
55+ years	1.04	0.690	(0.86–1.26)	0.68	0.004	(0.52–0.88)
Harmful drinking (ref.: AUDIT-C score < 8)	0.83	0.067	(0.69–1.01)	4.08	<0.001	(3.43–4.85)

Note: Adjusted for country and week of participation. ref.: reference. Missing values: 6.1%.

### Changes in tobacco use

Prevalence of tobacco users ranged between less than one-quarter in Kosovo (20.7%, 95% CI: 16.0, 26.4) and Moldova (23.6%, 95% CI: 17.0, 31.9) to more than half of the respondents in Romania (54.2%, 95% CI: 48.1, 60.3). About half of those using tobacco reported no change in their frequency of use. On average, no change in the frequency of tobacco use was observed in Armenia, Estonia, Georgia, Kosovo, Russia, Turkey and Ukraine (see Supplementary table S2). In the remaining countries, the regression analyses revealed a substantial increase in tobacco use. In other words, on average, more people tended to increase than decrease their tobacco use, except of Moldova, where the opposite was true.

With regard to the gender differences in the self-reported change of tobacco use (table 3), analysis indicated that women compared with men were at higher risk to report an increase vs. no change in their use, while there was no gender difference in reporting a decrease in use. In contrast to middle-aged adults, young adults were more likely to report both decreases and increases in their tobacco use compared with no change, and older adults were less likely to increase their tobacco use. Respondents who reported changes in their alcohol use were generally also more likely to report changes in their tobacco use, with a markedly higher risk of changing their substance use in the same direction. Specifically, respondents who reported decreases in alcohol use compared with no change were 3.6 times more likely to decrease their tobacco use than those reporting no change in tobacco use, and vice versa.

**Table 3:** Multinomial regression of self-reported decrease or increase in tobacco use compared with no change in tobacco use, *n *=* *3289

	Decrease vs. no change			Increase vs. no change		
	RRR	*P*	95% CI	RRR	*P*	95% CI
Gender: women (ref.: men)	1.13	0.247	(0.92–1.40)	1.35	<0.001	(1.14–1.60)
Age group (ref.: 35–54 years)						
18–34 years	1.80	<0.001	(1.43–2.25)	1.26	0.009	(1.06–1.49)
≥55 years	1.05	0.852	(0.67–1.62)	0.54	0.001	(0.38–0.77)
Overall change in alcohol use (ref.: no change)						
Decrease	3.61	<0.001	(2.83–4.62)	1.58	<0.001	(1.31–1.91)
Increase	1.79	<0.001	(1.32–2.43)	3.59	<0.001	(2.94–4.39)

Note: Adjusted for country and week of participation. ref.: reference. Missing values: 11.9%.

## Discussion

In a sample of more than 11 000 adults from 18 countries located in the eastern part of the WHO European Region, we examined changes in substance use during the COVID-19 pandemic. In most countries studied, about half of the respondents indicating past-year alcohol or tobacco use reported no changes in their substance use over the past 3 months. Overall, alcohol use seems to have decreased on average in most countries, while the opposite was true for tobacco. In-depth analysis revealed that, in our sample, women were less likely than men to decrease their alcohol use and more likely to increase their tobacco use compared with no change. With regard to age patterns, young respondents were more likely to report changes in the use of both substances and in both directions. Finally, our analysis showed that those who reported past-year harmful alcohol use had a 4-fold increased risk of increasing their alcohol use during the past three months.

### Study limitations

This study was the first comprehensive investigation to explore substance use changes in the eastern part of the WHO European Region in the course of the COVID-19 pandemic. Before placing our findings into the broader context, key limitations of this research should be outlined. First, our sample represents a convenience sample and thus, may not reflect the actual population of the countries studied (see Supplementary table S1), similar to other pan-European population-based surveys during COVID-19.^23^ To account for skewed sample distribution, we calculated survey weights that took into account national population distribution with regard to gender, age and educational attainment. However, an adequate geographical distribution of respondents within countries cannot be guaranteed, particularly for countries of the size of Russia, and a higher proportion is likely to stem from urban areas while rural areas will be inadequately covered. Additionally, only people having access to internet were able to participate.^23^ Second, self-reported measures of substance use are usually subject to underreporting^24^ and are often influenced by social-desirability bias.^25^ To validate our results, further data sources such as sales statistics on alcohol and tobacco are needed, but not yet available. Third, our study covers a period of more than 6 months in which different countries have experienced different conditions related to the COVID-19 pandemic, and the countries we consider have varied widely in the measures taken to contain the COVID-19 pandemic (see ^6^ for an overview of governmental responses to COVID-19 pandemic). To control for the timing of the survey as well as for national differences in the COVID-19 response, we included both indicators in multinomial regression analyses. Fourth, our study assessed changes in substance use in a cross-sectional design. Cohort and longitudinal studies would be favourable to evaluate changes over time, particularly in the medium and the long term. Fifth, we cannot rule out the possibility that changes in substance use have been caused by factors other than the COVID-19 pandemic.^26^ Finally, it is important to emphasize that while the described results contribute to a better understanding of self-reported changes in alcohol and tobacco use in the eastern part of the Region, they should be treated with caution especially in drawing any parallels to countries from Western and Central Europe. As shown in Supplementary tables S5 and S6 for alcohol use and Supplementary table S7 for tobacco use, there is a large variation between the analysed countries in terms of alcohol and tobacco key indicators, such as the prevalence of current drinkers or HED or the prevalence of tobacco use in the population, alcohol and tobacco use by sex as well as alcoholic beverage type and the proportion of unrecorded alcohol in total per capita consumption. For instance, with regard to alcohol, some of the included countries have the regionally lowest drinking levels, while others have the regionally and globally highest levels of alcohol per capita consumption. This diversity makes the interpretation of the overall results very challenging.

### Interpretation

In line with previous research on substance use changes during the pandemic, our findings suggest an average decline in the use of alcohol and an increase in tobacco use in countries of the eastern part of the WHO European Region.^9^^,^^10^^,^^14^^,^^17^^,^^27^ For alcohol, this trend was observed in all countries, except of Belarus, Latvia, Lithuania (see also ^27^), and Turkey. Researchers expected a decline in alcohol use within the general population, given the restrictions imposed to contain the spread of the virus,^5^ with measures taken at least temporarily in almost all countries to curb the pandemic.^6^ When comparing our findings to the results from another pan-European survey employing a similar methodology,^10^ the most pronounced decline in the current survey was found in the drinking frequency while the other survey found the strongest decline in the frequency of HED events. Although this comparison has not been statistically tested, there are indications that the decline in alcohol use in this study is somewhat different from that in the Western and Central European survey. The decline in the frequency of HED events was previously connected to fewer drinking opportunities that can involve heavy drinking, for example, the closure of bars and pubs or restrictions on social gatherings.^28^ In contrast, minor average declines in the current study might be related to a different drinking culture, where HED is less a social phenomenon but part of a regular drinking practice,^29^ or rather uncommon for religious reasons, as, for example, in Kosovo, where the majority of the population is Muslim. An alternative hypothesis could be the different timing of the surveys, as this one was conducted in the second half of 2020, when the COVID-19 pandemic had been present for more than half a year and the most severe COVID-19 measures like lockdowns and closing of drinking venues were partly lifted in some countries. There were also large differences in the taken response measures to the pandemic, as some countries introduced stricter lockdowns and restrictions that led to the closure of bars and restaurants, while other countries had a more laissez-faire approach.^6^ For instance, Belarus might be the only country from the sample where no self-isolation measures and lockdowns were introduced for the entire time of the pandemic, while others countries had some forms of restrictions in place.^30^ Notably, Latvia was the only country where, on average, the frequency of drinking was reported to be increased, while being one of the only countries that has loosened its alcohol control legislation during the COVID-19 pandemic by allowing alcohol sales and deliveries over the internet.^31–33^ More research is necessary to identify underlying mechanisms at play leading to the observed patterns of change.

Our findings revealed that younger adults were most likely to change their substance use, both for alcohol and for tobacco use. Young adults may have been particularly affected by the consequences of the pandemic, such as job losses,^34^ or some measures may have had a particular impact on their substance use, such as the restriction of social gatherings.^12^ This age group has also been found to have the highest levels of mental distress,^35^^,^^36^ with greater distress experiences being identified as a contributing factor to increased substance use.^10^ In line with prior research, we found women to be more likely to increase their tobacco use^10^^,^^13^ while being less likely to decrease their alcohol use as compared with men. As with young adults, research indicated that women face greater exposure to the adverse effects of the pandemic,^36^^,^^37^ which could be linked to increases (or less pronounced decreases) in substance use. Finally, people with harmful drinking prior to the pandemic were more likely to increase their drinking during the pandemic. This finding complements to the body of research pointing to a shift in the distribution of alcohol use during the COVID-19 pandemic,^17^^,^^38^ with alcohol use possibly decreasing in the general population^7–9^^,^^28^ while increasing among harmful alcohol users.^15^^,^^17^^,^^18^

## Conclusion

This is the first research exploring changes in substance use during the COVID-19 pandemic in countries of the eastern part of the WHO European Region. In the countries studied, every second respondent using alcohol or tobacco reported having changed their substance use in the last 3 months. Changes in alcohol and tobacco use seem to happen across substances, with women, young adults and past-year harmful users being identified as more vulnerable to change. The results point to a growing health inequality when factoring the health consequences of alcohol and tobacco use.^39^^,^^40^

## Supplementary data

Supplementary data are available at *EURPUB* online.

## Supplementary Material

ckac011_Supplementary_Data
