# Supplementary material for: Self-reported changes in alcohol and tobacco use during COVID-19: findings from the eastern part of WHO European Region
Source: Eur J Public Health. 2022 Feb 7;32(3):474–80. doi: 10.1093/eurpub/ckac011 (PMC9159328; doi:10.1093/eurpub/ckac011)
Supplement: ckac011_Supplementary_Data [file ckac011_supplementary_data.docx]

# Supplementary material

**Title:** Self-reported changes in alcohol and tobacco use in the course of the COVID-19 pandemic: findings from 18 countries in the eastern part of the WHO European Region

**Authors:** Carolin Kilian^†^, Maria Neufeld^†^, Jakob Manthey, Sophiko Alavidze, Anastacia Bobrova, Orna Baron-Epel, Merita Berisha, Rabia Bilici, Kairat Davletov, Laura Isajeva, Fatma Kantaş Yılmaz, Tatsiana Karatkevich, Alibek Mereke, Sanja Musić Milanović, Kristine Galstyan, Ljiljana Muslić, Michail Okoliyski, Zana Shabani, Mindaugas Štelemėkas, Lela Sturua, Sharon R. Sznitman, Başak Ünübol, Carina Ferreira-Borges, Jürgen Rehm

^†^ shared first authors

**Supplement S1: Calculation of survey weights**

Survey weights were calculated as the inverse probability for taking the survey, calculated for 18 strata per country. These 18 strata were defined by the variables gender (male, female), age (18–34, 35–54, ≥55) and education (lower, middle, higher). The actual population distribution of these strata was obtained from Eurostat [1] and national statistical offices. For Russia, data were taken from the Russian census in 2010 [2] which was adapted to the total population estimate from 2019. The same procedure was applied to Ukraine considering the most recent census data from 2001 [3] and population estimates from 2020 [4], and to Kosovo^[[1]](#footnote-1)^ using census data from 2011 [5] and population estimates from 2019 [6]. In the case of Kazakhstan, the average population distribution in strata of gender and age were available for the year 2020 [7], while the population distribution by educational attainment was estimated based on the two neighbouring countries Kyrgyzstan and Russia. In Georgia, the average population distributions for the 18 strata were estimated using the information from Armenia, Turkey, and Russia which were applied to the distribution of the country’s population by gender and age group in 2020 [8].

As no population statistics were available on individuals with “other” gender, persons of “other” gender were randomly assigned to another gender within the same age- and education-strata for calculating the weights.

Strata were collapsed within each gender if the resulting weight exceeded 10. Out of all 38 country–gender combinations, no stratum was collapsed in 7 cases due to weights in all strata not exceeding 10. For the other country–gender combinations, the best fitting solution in collapsing strata was selected from the following options:

First–order: collapsing concerned stratum with 1 direct stratum neighbour; e.g., A with B or D

Second–order: collapsing concerned stratum with 3 stratum neighbours; e.g., A with B, D and E

Third–order: collapsing concerned stratum with 6 stratum neighbours; e.g., A with B, D, E, G and H

|  | *Education 1* | *Education 2* | *Education 3* |
| --- | --- | --- | --- |
| *Age 1* | A | B | C |
| *Age 2* | D | E | F |
| *Age 3* | G | H | I |

For country–gender combinations for which only 1 stratum had to be collapsed, the best fitting solution was a first–order collapse with the resulting weight being lower than 10. If multiple first–order collapses had a weight below 10, the lowest was chosen. If no first–order collapse had a weight lower 10, a second–order and subsequently a third–order solution was considered. The same criteria (a weight lower 10 and choosing the solution with the lowest weight for multiple collapses) were applied in determining the best fitting solution.

For country–gender combinations for which more than 1 stratum had to be collapsed, the overlap of best fitting strata collapses was considered. The most economical solution was chosen, i.e., order 2 was preferred over order 3 combinations. In addition, collapsing 2 (or 3) age groups was preferred over collapsing 2 (or 3) education groups. If this allowed several options, the smaller weight was chosen. Third order solutions were applied in 18 country–gender combinations (Armenia–Males, Armenia–Females, Belarus–Males, Bulgaria–Males, Georgia–Males, Georgia–Females, Kazakhstan–Females, Kazakhstan–Males, Kosovo–Females, Kosovo–Males, Kyrgyzstan–Males, Latvia–Females, Lithuania–Males, Montenegro–Males, Romania–Males, Romania–Females, Turkey–Females, Ukraine–Males).

**Table S1. Unweighted sample characteristics (N = 11,295) and actual population characteristics by country.**

| Country | | Number | Gender (% women)^a^ | Age | | | Educational attainment: High school or lower |
| --- | --- | --- | --- | --- | --- | --- | --- |
|  |  |  |  | 18-34 years | 35-54 years | 55+ years |  |
| Armenia | Unweighted sample | 366 | 52.7 (47.6, 57.8) | 54.6 (49.5, 59.7) | 38.3 (33.4, 43.3) | 7.1 (4.9, 10.2) | 5.5 (3.6, 8.3) |
|  | Actual population | 2,192,355 | 52.8 | 30.7 | 34.7 | 34.6 | 62.1 |
| Belarus | Unweighted sample | 516 | 61.6 (57.4, 65.7) | 43 (38.8, 47.3) | 45.7 (41.5, 50.1) | 11.2 (8.8, 14.3) | 8.7 (6.6, 11.5) |
|  | Actual population | 6,981,600 | 53.3 | 30.1 | 38.1 | 31.8 | 53.5 |
| Bulgaria | Unweighted sample | 600 | 73.2 (69.5, 76.6) | 27.5 (24.1, 31.2) | 61.5 (57.5, 65.3) | 11 (8.7, 13.8) | 12.2 (9.8, 15) |
|  | Actual population | 5,167,400 | 50.8 | 25.6 | 39.0 | 35.4 | 75.5 |
| Croatia | Unweighted sample | 554 | 74.9 (71.1, 78.3) | 39.9 (35.9, 44) | 51.6 (47.5, 55.8) | 8.5 (6.4, 11.1) | 29.2 (25.6, 33.2) |
|  | Actual population | 2,998,000 | 50.8 | 27.6 | 36.9 | 35.6 | 77.7 |
| Estonia | Unweighted sample | 347 | 77.5 (72.8, 81.6) | 36 (31.1, 41.2) | 55.6 (50.3, 60.8) | 8.4 (5.9, 11.8) | 17.6 (13.9, 21.9) |
|  | Actual population | 940,000 | 51.5 | 29.2 | 38.1 | 32.8 | 62.2 |
| Georgia^b^ | Unweighted sample | 604 | 78.1 (74.7, 81.3) | 46.9 (42.9, 50.8) | 40.4 (36.6, 44.4) | 12.7 (10.3, 15.7) | 8.4 (6.5, 10.9) |
|  | Actual population | 2,609,651 | 51.9 | 31.5 | 36.8 | 31.7 | . |
| Israel | Unweighted sample | 454 | 52.2 (47.6, 56.8) | 40.7 (36.3, 45.3) | 36.6 (32.3, 41.1) | 22.7 (19.1, 26.8) | 29.1 (25.1, 33.4) |
|  | Actual population | 3,869,948 | 53.6 | 32.4 | 39.5 | 28.0 | 59.4 |
| Kazakhstan^b^ | Unweighted sample | 457 | 73.1 (68.8, 77) | 66.7 (62.3, 70.9) | 28.2 (24.3, 32.5) | 5 (3.4, 7.5) | 6.3 (4.4, 9) |
|  | Actual population | 13,621,541 | 52.1 | 34.4 | 45.5 | 20.1 | . |
| Kosovo | Unweighted sample | 499 | 73.3 (69.3, 77) | 84.8 (81.3, 87.7) | 12.4 (9.8, 15.6) | 2.8 (1.7, 4.7) | 43.9 (39.6, 48.3) |
|  | Actual population | 1,782,115 | 48.6 | 42.4 | 39.3 | 18.3 | 91.5 |
| Kyrgyzstan | Unweighted sample | 471 | 73.1 (68.8, 88.6) | 42.3 (37.9, 46.8) | 40.3 (36, 44.8) | 17.4 (14.2, 21.1) | 38.2 (33.9, 42.7) |
|  | Actual population | 4,067,308 | 51.3 | 45.3 | 34.7 | 19.9 | 90.4 |
| Latvia | Unweighted sample | 1988 | 55.9 (53.7, 58.1) | 50.9 (48.7, 53) | 40.8 (38.7, 43) | 8.3 (7.2, 9.6) | 19.1 (17.4, 20.9) |
|  | Actual population | 1,346,400 | 52.9 | 27.7 | 37.9 | 34.4 | 68.3 |
| Lithuania | Unweighted sample | 577 | 82.5 (79.2, 85.4) | 56.3 (52.2, 60.3) | 31.9 (28.2, 35.8) | 11.8 (9.4, 14.7) | 19.8 (16.7, 23.2) |
|  | Actual population | 2,017,100 | 52.4 | 29.1 | 36.8 | 34.1 | 62.6 |
| Moldova | Unweighted sample | 660 | 73.8 (70.3, 77) | 62.4 (58.7, 66) | 31.4 (27.9, 35) | 6.2 (4.6, 8.3) | 18.5 (15.7, 21.6) |
|  | Actual population | 1,631,770 | 51.4 | 33.3 | 43.0 | 23.7 | 85.6 |
| Montenegro | Unweighted sample | 471 | 64.5 (60.1, 68.7) | 62.6 (58.2, 66.9) | 33.8 (29.6, 38.2) | 3.6 (2.3, 5.7) | 16.1 (13.1, 19.7) |
|  | Actual population | 446,732 | 50.6 | 34.5 | 38.3 | 27.2 | 78.0 |
| Romania | Unweighted sample | 1079 | 49.7 (46.7, 52.7) | 51.7 (48.7, 54.7) | 46.4 (43.5, 49.4) | 1.9 (1.2, 2.9) | 17.5 (15.4, 19.9) |
|  | Actual population | 14,192,000 | 50.4 | 27.5 | 40.9 | 31.6 | 84.5 |
| Russia | Unweighted sample | 576 | 58.9 (54.8, 62.8) | 54.9 (50.8, 58.9) | 35.9 (32.1, 39.9) | 9.2 (7.1, 11.8) | 25 (21.6, 28.7) |
|  | Actual population | 146,764,653 | 55.5 | 34.1 | 36.2 | 29.8 | 61.2 |
| Turkey | Unweighted sample | 466 | 66.7 (62.3, 70.9) | 70.4 (66.1, 74.4) | 26.4 (22.6, 30.6) | 3.2 (1.9, 5.3) | 26.8 (23, 31) |
|  | Actual population | 54,283,512 | 49.5 | 37.4 | 41.3 | 22.1 | 81.6 |
| Ukraine | Unweighted sample | 610 | 73.6 (70, 77) | 35.4 (31.7, 39.3) | 52.3 (48.3, 56.2) | 12.3 (9.9, 15.1) | 10 (7.9, 12.6) |
|  | Actual population | 35,603,935 | 54.0 | 35.6 | 35.3 | 29.1 | 84.9 |

^a^ Proportion of those reporting other gender was < 1% in all countries. ^b^ For Georgia and Kazakhstan, population data were only available by gender and age group (see Supplement S1). 95% confidence intervals are presented in brackets.

**Table S2. Comparison of key characteristics between complete cases and imputed cases.**

|  |  | Complete cases  (95% CI) | Imputed cases  (95% CI) | |
| --- | --- | --- | --- | --- |
| **Number of observations (*n*)** | | 10,485 | | 294 |
| **Gender (%)** | Women | 45.3 (43.5, 47.1) | | 38.9 (29.1, 49.8) |
|  | Men | 54.4 (52.6, 56.2) | | 61.1 (50.2, 70.9) |
|  | Other | 0.3 (0.1, 0.7) | | 0 |
| **Age groups (%)** | 18-34 years | 46.5 (44.7, 48.3) | | 43.9 (33.8, 54.5) |
|  | 35-54 years | 40.9 (39.1, 42.8) | | 39.4 (29.6, 50.2) |
|  | ≥ 55 years | 12.6 (11.4, 13.8) | | 16.7 (11.0, 24.5) |
| **Educational attainment (%)** | High school or lower | 67.7 (66.3, 69.1) | | 72.1 (63.6, 79.2) |
|  | Any education beyond high school | 32.3 (30.9, 33.7) | | 27.9 (20.8, 36.4) |

Belarus has been excluded as no data was collected on the past-year quantity of alcohol consumed per drink day and the past-year frequency of heavy episodic drinking. CI = confidence intervals. All data weighted.

**Table S3. Changes in substance use by country.**

| **Country** |  | **Alcohol: overall use** | **Alcohol: Drinking frequency** | **Alcohol: Drinking quantity** | **Alcohol: Frequency of HED** | **Tobacco** |
| --- | --- | --- | --- | --- | --- | --- |
| Armenia | n | 285 | 285 | 285 | 285 | 111 |
|  | Decrease | 42.8 (31.2, 55.2) | 26.8 (17.3, 39.0) | 24.3 (15.0, 36.8) | 27.7 (17.5, 40.9) | 12.7 (3.8, 34.8) |
|  | No change | 49.4 (37.8, 61.2) | 64.5 (52.4, 75.0) | 71.9 (59.7, 81.5) | 69.5 (56.6, 80.0) | 59.4 (39.1, 76.9) |
|  | Increase | 7.8 (3.2, 17.7) | 8.7 (4.0, 17.9) | 3.8 (1.7, 8.4) | 2.8 (1.1, 6.9) | 27.9 (14.5, 47.0) |
|  | Mean change^1^ | -0.17 (*p* < .001) | -0.17 (*p* = .002) | -0.18 (*p* < .001) | -0.17 (*p* < .001) | 0.07 (*p* = .496) |
| Belarus | n | 428 | 428 | 428 | 428 | 118 |
|  | Decrease | 17.2 (12.1, 23.7) | 11.6 (7.9, 16.8) | 14.5 (10.0, 20.6) | 14.1 (9.7, 20.2) | 16.1 (7.0, 32.9) |
|  | No change | 65.9 (57.6, 73.4) | 73.3 (65.8, 79.7) | 78.7 (71.8, 84.4) | 77.1 (69.5, 83.2) | 54.6 (38.9, 69.5) |
|  | Increase | 16.9 (11.2, 24.6) | 15.0 (9.9, 22.1) | 6.7 (3.7, 11.9) | 8.8 (4.8, 15.6) | 29.3 (17.9, 44.1) |
|  | Mean change^1^ | -0.04 (*p* = .122) | 0.01 (*p* = .650) | -0.06 (*p* = .011) | -0.05 (*p* = .050) | 0.10 (*p* = .131) |
| Bulgaria | n | 531 | 531 | 531 | 531 | 258 |
|  | Decrease | 29.1 (22.6, 36.4) | 24.4 (18.4, 31.7) | 19.2 (13.8, 26.1) | 22.8 (16.8, 30.2) | 14.6 (9.1, 22.6) |
|  | No change | 49.2 (41.7, 56.8) | 55.8 (48.3, 63.1) | 65.6 (58.1, 72.4) | 65.9 (58.2, 72.9) | 45.3 (34.5, 56.6) |
|  | Increase | 21.7 (16.3, 28.4) | 19.8 (14.7, 26.0) | 15.2 (10.7, 21.2) | 11.3 (7.2, 17.2) | 40.1 (29.6, 51.5) |
|  | Mean change^1^ | -0.09 (*p* =.021) | -0.06 (*p* = .141) | -0.07 (*p* = .062) | -0.13 (*p* = .002) | 0.19 (*p* = .001) |
| Croatia | N | 453 | 453 | 453 | 453 | 239 |
|  | Decrease | 43.1 (35.2, 51.3) | 34.4 (27.2, 42.3) | 22.1 (16.4, 29.0) | 28.6 (22.1, 36.2) | 8.3 (4.8, 13.9) |
|  | No change | 44.0 (35.9, 52.4) | 55.2 (47.2, 63.0) | 67.3 (59.5, 74.1) | 61.4 (53.4, 68.9) | 44.4 (33.5, 55.9) |
|  | Increase | 12.9 (8.7, 18.8) | 10.4 (7.2, 14.9) | 10.7 (6.8, 16.4) | 9.9 (6.2, 15.5) | 47.3 (36.5, 58.3) |
|  | Mean change^1^ | -0.17 (*p* < .001) | -0.24 (*p* < .001) | -0.10 (*p* = .005) | -0.17 (*p* < .001) | 0.29 (*p* < .001) |
| Estonia | n | 313 | 313 | 313 | 313 | 87 |
|  | Decrease | 40.5 (30.9, 51.0) | 35.1 (25.9, 45.6) | 28.3 (19.8, 38.7) | 28.6 (19.9, 39.1) | 19.3 (9.6, 35.1) |
|  | No change | 40.1 (30.4, 50.7) | 50.3 (40.1, 60.4) | 55.0 (44.7, 64.9) | 61.7 (51.1, 71.2) | 49.9 (33.3, 66.5) |
|  | Increase | 19.3 (12.9, 28.0) | 14.6 (9.2, 22.4) | 16.7 (10.5, 25.5) | 9.8 (5.2, 17.6) | 30.8 (17.3, 48.5) |
|  | Mean change^1^ | -0.15 (*p* = .001) | -0.19 (*p* = .001) | -0.11 (*p* = .016) | -0.16 (*p* = .001) | 0.05 (*p* = .592) |
| Georgia | n | 404 | 404 | 404 | 404 | 219 |
|  | Decrease | 50.2 (41.0, 59.3) | 42.3 (33.6, 51.5) | 39.1 (30.6, 48.2) | 36.5 (28.2, 45.8) | 19.9 (11.8, 31.7) |
|  | No change | 41.0 (32.3, 50.2) | 48.9 (40.0, 58.0) | 57.1 (48.0, 65.8) | 60.7 (51.4, 69.3) | 48.6 (37.1, 60.3) |
|  | Increase | 8.9 (4.7, 16.0) | 8.8 (4.7, 15.6) | 3.8 (1.6, 8.9) | 2.7 (0.9, 8.3) | 31.4 (21.8, 43.0) |
|  | Mean change^1^ | -0.29 (*p* < .001) | -0.30 (*p* < .001) | -0.30 (*p* < .001) | -0.27 (*p* < .001) | 0.06 (*p* = .397) |
| Israel | n | 374 | 374 | 374 | 374 | 157 |
|  | Decrease | 29.0 (23.4, 35.3) | 21.5 (16.6, 27.4) | 17.7 (13.4, 23.1) | 19.0 (14.5, 24.6) | 8.6 (5.1, 14.4) |
|  | No change | 47.3 (40.6, 54.0) | 54.8 (48.0, 61.4) | 62.5 (55.7, 68.9) | 70.7 (64.2, 76.4) | 57.5 (47.5, 66.9) |
|  | Increase | 23.7 (18.2, 30.3) | 23.7 (18.2, 30.2) | 19.7 (14.5, 26.2) | 10.3 (6.7, 15.6) | 33.9 (25.3, 43.7) |
|  | Mean change^1^ | -0.06 (*p* = .032) | -0.04 (*p* = .197) | -0.04 (*p* = .211) | -0.09 (*p* = .001) | 0.18 (*p* < .001) |
| Kazakhstan | n | 234 | 234 | 234 | 234 | 95 |
|  | Decrease | 41.7 (31.1, 53.2) | 35.9 (25.9, 47.3) | 31.5 (21.9, 43.0) | 28.9 (19.5, 40.7) | 29.8 (16.7, 47.4) |
|  | No change | 38.8 (27.9, 51.1) | 47.1 (36.1, 58.3) | 54.8 (43.7, 65.5) | 58.7 (47.4, 69.1) | 44.9 (29.9, 60.9) |
|  | Increase | 19.5 (13.4, 27.4) | 17.0 (11.6, 24.4) | 13.7 (8.8, 20.5) | 12.4 (7.8, 19.1) | 25.2 (15.2, 39.0) |
|  | Mean change^1^ | -0.18 (*p* = .003) | -0.19 (*p* = .004) | -0.18 (*p* = .005) | -0.18 (*p* = .004) | -0.01 (*p* = .908) |
| Kosovo^[[2]](#footnote-2)^ | n | 176 | 176 | 176 | 176 | 104 |
|  | Decrease | 74.0 (60.9, 83.8) | 72.6 (60.0, 82.3) | 57.3 (44.4, 69.3) | 30.4 (19.8, 43.5) | 24.8 (14.6, 38.8) |
|  | No change | 16.6 (9.0, 28.8) | 20.9 (12.4, 33.0) | 34.3 (23.3, 47.2) | 66.9 (53.7, 77.8) | 29.3 (18.3, 43.5) |
|  | Increase | 9.4 (4.0, 20.3) | 6.5 (2.5, 16.0) | 8.4 (3.5, 18.8) | 2.8 (0.7, 10.6) | 45.9 (32.5, 59.9) |
|  | Mean change^1^ | -0.44 (*p* < .001) | -0.60 (*p* < .001) | -0.44 (*p* < .001) | -0.27 (*p* < .001) | 0.13 (*p* = .167) |
| Kyrgyzstan | n | 139 | 139 | 139 | 139 | 65 |
|  | Decrease | 50.8 (32.2, 69.2) | 41.0 (24.6, 59.6) | 32.3 (18.7, 49.6) | 38.1 (22.2, 57.2) | 20.7 (8.3, 42.9) |
|  | No change | 34.2 (18.4, 54.6) | 46.2 (28.9, 64.4) | 56.0 (38.4, 72.2) | 52.2 (33.8, 69.9) | 31.9 (14.1, 57.3) |
|  | Increase | 15.0 (5.8, 33.6) | 12.9 (4.8, 30.3) | 11.7 (4.2, 28.6) | 9.7 (2.7, 29.2) | 47.3 (26.1, 69.6) |
|  | Mean change^1^ | -0.17 (*p* = .025) | -0.13 (*p* = .167) | -0.16 (*p* = .028) | -0.22 (*p* = .011) | 0.12 (*p* = .373) |
| Latvia | n | 1,738 | 1,738 | 1,738 | 1,738 | 727 |
|  | Decrease | 25.0 (21.7, 28.7) | 19.3 (16.4, 22.7) | 17.1 (14.3, 20.4) | 20.4 (17.3, 23.9) | 14.5 (10.6, 19.5) |
|  | No change | 40.5 (36.5, 44.6) | 47.8 (43.7, 51.8) | 59.9 (55.8, 63.8) | 56.1 (52.0, 60.1) | 39.1 (33.4, 45.1) |
|  | Increase | 34.4 (30.6, 38.5) | 32.9 (29.2, 36.8) | 23.0 (19.7, 26.7) | 23.5 (20.1, 27.3) | 46.4 (40.5, 52.4) |
|  | Mean change^1^ | 0.03 (*p* = .172) | 0.07 (*p* = .002) | 0.02 (*p* = .387) | -0.01 (*p* = .712) | 0.26 (*p* < .001) |
| Lithuania | n | 475 | 475 | 475 | 475 | 144 |
|  | Decrease | 43.8 (34.0, 54.1) | 34.6 (25.5, 45.0) | 25.5 (17.7, 35.2) | 27.6 (19.4, 37.7) | 8.9 (3.3, 21.6) |
|  | No change | 31.4 (23.1, 41.1) | 42.1 (32.7, 52.0) | 53.3 (43.2, 63.1) | 53.9 (43.6, 63.8) | 55.1 (37.9, 71.2) |
|  | Increase | 24.8 (16.9, 34.7) | 23.3 (16.0, 32.7) | 21.3 (13.8, 31.4) | 18.5 (11.4, 28.5) | 36.0 (21.4, 53.7) |
|  | Mean change^1^ | -0.08 (*p* = .201) | -0.11 (*p* = .125) | -0.06 (*p* = .350) | -0.06 (*p* = .322) | 0.23 (*p* = .005) |
| Moldova | n | 512 | 512 | 512 | 512 | 130 |
|  | Decrease | 61.0 (51.6, 69.6) | 52.9 (43.5, 62.1) | 45.2 (35.9, 54.9) | 40.9 (31.5, 51.0) | 40.6 (24.1, 59.6) |
|  | No change | 29.8 (22.3, 38.5) | 36.6 (28.4, 45.7) | 49.6 (40.2, 59.0) | 52.8 (43.0, 62.3) | 44.8 (27.7, 63.2) |
|  | Increase | 9.2 (4.8, 16.9) | 10.5 (5.9, 18.0) | 5.2 (2.2, 11.9) | 6.3 (2.6, 14.6) | 14.6 (6.8, 28.6) |
|  | Mean change^1^ | -0.35 (*p* < .001) | -0.37 (*p* < .001) | -0.36 (*p* < .001) | -0.32 (*p* < .001) | -0.28 (*p* = .008) |
| Montenegro | n | 388 | 388 | 388 | 388 | 198 |
|  | Decrease | 46.1 (34.7, 57.8) | 45.8 (34.7, 57.4) | 29.2 (19.4, 41.4) | 26.2 (17.8, 36.7) | 12.9 (6.0, 25.5) |
|  | No change | 31.1 (21.3, 42.9) | 33.4 (23.6, 44.9) | 50.9 (39.5, 62.2) | 58.7 (47.3, 69.3) | 35.4 (21.8, 51.9) |
|  | Increase | 22.8 (15.1, 33.1) | 20.8 (13.6, 30.4) | 19.9 (12.6, 30.0) | 15.1 (8.6, 25.2) | 51.7 (36.1, 67.0) |
|  | Mean change^1^ | -0.15 (*p* = .012) | -0.26 (*p* = .001) | -0.09 (*p* = .235) | -0.10 (*p* = .087) | 0.31 (*p* = .001) |
| Romania | n | 993 | 993 | 993 | 993 | 495 |
|  | Decrease | 47.7 (41.3, 54.1) | 37.3 (31.4, 43.6) | 30.2 (24.7, 36.3) | 40.3 (34.2, 46.8) | 20.8 (14.7, 28.5) |
|  | No change | 29.2 (23.8, 35.3) | 38.5 (32.6, 44.8) | 51.3 (45.0, 57.6) | 44.8 (38.6, 51.2) | 24.5 (18.0, 32.5) |
|  | Increase | 23.1 (18.2, 28.9) | 24.2 (19.2, 29.9) | 18.5 (14.1, 23.9) | 14.9 (10.9, 20.0) | 54.7 (46.2, 63.0) |
|  | Mean change^1^ | -0.16 (*p* < .001) | -0.13 (*p* = .001) | -0.11 (*p* < .001) | -0.23 (*p* < .001) | 0.27 (*p* < .001) |
| Russia | n | 457 | 457 | 457 | 457 | 238 |
|  | Decrease | 41.0 (34.4, 48.0) | 35.2 (29.0, 42.0) | 30.3 (24.5, 36.8) | 34.5 (28.1, 41.4) | 19.3 (12.1, 29.2) |
|  | No change | 37.2 (30.7, 44.2) | 46.7 (39.8, 53.8) | 57.9 (51.0, 64.5) | 51.2 (44.1, 58.2) | 45.2 (35.1, 55.6) |
|  | Increase | 21.8 (16.0, 29.0) | 18.1 (13.5, 23.7) | 11.8 (8.3, 16.5) | 14.4 (9.4, 21.4) | 35.6 (26.4, 45.9) |
|  | Mean change^1^ | -0.17 (*p* < .001) | -0.15 (*p* < .001) | -0.17 (*p* < .001) | -0.18 (*p* < .001) | 0.08 (*p* = .206) |
| Turkey | n | 195 | 195 | 195 | 195 | 147 |
|  | Decrease | 35.2 (24.1, 48.2) | 31.8 (21.2, 44.7) | 26.3 (16.3, 39.5) | 19.5 (10.4, 33.5) | 26.9 (15.7, 42.1) |
|  | No change | 41.4 (29.2, 54.7) | 46.9 (34.5, 59.6) | 59.3 (46.3, 71.2) | 68.6 (54.5, 79.9) | 43.1 (28.2, 59.5) |
|  | Increase | 23.4 (14.6, 35.4) | 21.3 (12.9, 33.1) | 14.3 (7.5, 25.7) | 11.9 (5.4, 24.3) | 30.0 (18.6, 44.5) |
|  | Mean change^1^ | -0.11 (*p* = .065) | -0.12 (*p* = .079) | -0.14 (*p* = .029) | -0.08 (*p* = .191) | 0.02 (*p* = .707) |
| Ukraine | n | 527 | 527 | 527 | 527 | 156 |
|  | Decrease | 34.1 (25.2, 44.3) | 25.4 (17.7, 35.1) | 19.1 (13.3, 26.5) | 25.4 (17.7, 35.0) | 18.1 (8.7, 33.9) |
|  | No change | 52.2 (42.0, 62.3) | 60.0 (49.9, 69.3) | 76.4 (68.6, 82.7) | 71.7 (62.1, 79.6) | 44.8 (27.8, 63.2) |
|  | Increase | 13.7 (8.5, 21.3) | 14.6 (9.3, 22.2) | 4.6 (2.7, 7.6) | 3.0 (1.6, 5.5) | 37.1 (22.2, 55.0) |
|  | Mean change^1^ | -0.14 (*p* < .001) | -0.11 (*p* = .009) | -0.12 (*p* < .001) | -0.19 (*p* < .001) | 0.10 (*p* = .271) |

Note: ^1^Mean changes reflect the results of the country-specific regression analyses, indicated by the y-axis intercept and its significance. A negative number indicates an average decrease and a positive an average increase in substance use. 95% confidence intervals are presented in brackets unless otherwise stated.

**Table S4.** **Sensitivity analysis: changes in substance use by country, excluding respondents with missing data for past-year quantity of pure alcohol consumed per drink day or past-year frequency of heavy episodic drinking.**

| **Country** |  | **Alcohol: overall use** | **Alcohol: Drinking frequency** | **Alcohol: Drinking quantity** | **Alcohol: Frequency of HED** |
| --- | --- | --- | --- | --- | --- |
| Armenia | n | 271 | 281 | 280 | 271 |
|  | Decrease | 44.9 (32.8, 57.6) | 27.6 (17.5, 40.8) | 24.8 (14.9, 38.4) | 29.1 (18.1, 43.1) |
|  | No change | 46.6 (35.2, 58.4) | 62.8 (50.0, 74.0) | 71.0 (57.9, 81.4) | 68.0 (54.3, 79.1) |
|  | Increase | 8.5 (3.5, 19.3) | 9.6 (4.3, 19.8) | 4.2 (1.8, 9.3) | 3.0 (1.1, 7.6) |
| Bulgaria | n | 526 | 533 | 531 | 527 |
|  | Decrease | 29.4 (22.9, 36.8) | 24.7 (18.6, 32.0) | 19.4 (13.9, 26.4) | 23.1 (17.1, 30.5) |
|  | No change | 48.5 (41.0, 56.1) | 55.3 (47.7, 62.6) | 65.1 (57.6, 72.0) | 65.4 (57.7, 72.5) |
|  | Increase | 22.0 (16.5, 28.8) | 20 (15.0, 26.3) | 15.4 (10.8, 21.5) | 11.5 (7.3, 17.5) |
| Croatia | N | 447 | 454 | 455 | 449 |
|  | Decrease | 42.9 (35.0, 51.3) | 34.8 (27.5, 42.8) | 22.3 (16.6, 29.4) | 28.4 (21.8, 36.0) |
|  | No change | 44.0 (35.8, 52.5) | 54.7 (46.5, 62.6) | 66.9 (59.1, 73.9) | 61.6 (53.5, 69.1) |
|  | Increase | 13.1 (8.8, 19.0) | 10.5 (7.2, 15.1) | 10.8 (6.8, 16.6) | 10.0 (6.3, 15.7) |
| Estonia | n | 313 | 320 | 321 | 314 |
|  | Decrease | 40.5 (30.9, 51.0) | 35.1 (25.9, 45.6) | 28.3 (19.8, 38.7) | 28.6 (19.9, 39.1) |
|  | No change | 40.1 (30.4, 50.7) | 50.3 (40.1, 60.4) | 55 (44.7, 64.9) | 61.7 (51.1, 71.2) |
|  | Increase | 19.3 (12.9, 28.0) | 14.6 (9.2, 22.4) | 16.7 (10.5, 25.5) | 9.8 (5.2, 17.6) |
| Georgia | n | 394 | 409 | 408 | 395 |
|  | Decrease | 50.6 (41.2, 59.8) | 43.0 (34.1, 52.4) | 39.8 (31.1, 49.1) | 36.5 (27.9, 45.9) |
|  | No change | 40.2 (31.5, 49.6) | 47.8 (38.7, 57.1) | 56.4 (47, 65.3) | 60.7 (51.2, 69.4) |
|  | Increase | 9.2 (4.9, 16.6) | 9.1 (4.9, 16.3) | 3.9 (1.6, 9.2) | 2.9 (0.9, 8.7) |
| Israel | n | 365 | 365 | 365 | 365 |
|  | Decrease | 29.1 (23.4, 35.5) | 21.3 (16.3, 27.4) | 17.9 (13.4, 23.3) | 19.1 (14.5, 24.7) |
|  | No change | 46.5 (39.8, 53.3) | 54.4 (47.5, 61.1) | 62.0 (55.0, 68.4) | 70.3 (63.7, 76.1) |
|  | Increase | 24.4 (18.8, 31.1) | 24.3 (18.6, 30.9) | 20.2 (14.9, 26.9) | 10.6 (6.9, 16.1) |
| Kazakhstan | n | 222 | 230 | 228 | 225 |
|  | Decrease | 37.0 (27.2, 48.0) | 30.8 (21.9, 41.4) | 26.9 (18.6, 37.2) | 23.2 (15.3, 33.6) |
|  | No change | 42.3 (30.9, 54.6) | 50.3 (39.2, 61.4) | 58.9 (48.0, 69.0) | 63.7 (52.9, 73.4) |
|  | Increase | 20.7 (14.2, 29.1) | 18.9 (12.8, 26.9) | 14.2 (9.1, 21.6) | 13.0 (8.1, 20.3) |
| Kosovo^[[3]](#footnote-3)^ | n | 158 | 164 | 163 | 160 |
|  | Decrease | 77.5 (63.8, 87.1) | 75.1 (61.7, 84.9) | 58.6 (44.5, 71.4) | 33.8 (21.8, 48.4) |
|  | No change | 14.2 (7.0, 26.9) | 17.5 (9.4, 30.1) | 33.6 (22.0, 47.7) | 62.8 (48.3, 75.3) |
|  | Increase | 8.3 (3.2, 20.0) | 7.4 (2.8, 18.3) | 7.8 (2.9, 19.1) | 3.3 (0.8, 12.6) |
| Kyrgyzstan | n | 132 | 142 | 147 | 135 |
|  | Decrease | 54.3 (34.6, 72.8) | 43.2 (25.9, 62.4) | 33.4 (19.0, 51.9) | 40.2 (23, 60.3) |
|  | No change | 29.4 (14.6, 50.4) | 42.9 (25.8, 62.0) | 53.7 (35.3, 71.1) | 49.1 (30.3, 68.1) |
|  | Increase | 16.3 (6.2, 36.2) | 13.9 (5.1, 32.5) | 12.9 (4.6, 31.3) | 10.7 (2.9, 32.2) |
| Latvia | n | 1,730 | 1,754 | 1,750 | 1,730 |
|  | Decrease | 25.0 (21.7, 28.6) | 19.3 (16.3, 22.6) | 17.1 (14.3, 20.4) | 20.4 (17.3, 23.9) |
|  | No change | 40.5 (36.5, 44.6) | 47.7 (43.6, 51.8) | 59.7 (55.6, 63.7) | 56.1 (51.9, 60.1) |
|  | Increase | 34.6 (30.8, 38.6) | 33.1 (29.3, 37.0) | 23.2 (19.8, 26.9) | 23.6 (20.2, 27.4) |
| Lithuania | n | 467 | 481 | 483 | 470 |
|  | Decrease | 44.9 (34.9, 55.4) | 36.9 (27.4, 47.6) | 27.8 (19.5, 38.1) | 27.8 (19.4, 38.0) |
|  | No change | 29.9 (21.8, 39.3) | 37.6 (28.7, 47.4) | 52.8 (42.6, 62.8) | 53.8 (43.4, 63.9) |
|  | Increase | 25.2 (17.0, 35.6) | 25.5 (17.5, 35.6) | 19.4 (12.1, 29.5) | 18.5 (11.1, 29.1) |
| Moldova | n | 489 | 506 | 505 | 494 |
|  | Decrease | 59.3 (49.6, 68.3) | 53.7 (44.1, 63.1) | 45.6 (36, 55.5) | 38.4 (28.9, 48.7) |
|  | No change | 30.8 (23.0, 39.9) | 35.1 (26.9, 44.2) | 48.8 (39.2, 58.4) | 54.9 (44.7, 64.6) |
|  | Increase | 9.9 (5.2, 18.0) | 11.2 (6.3, 19.1) | 5.6 (2.3, 12.8) | 6.8 (2.8, 15.6) |
| Montenegro | n | 384 | 399 | 394 | 387 |
|  | Decrease | 46.1 (34.7, 57.9) | 45.9 (34.7, 57.5) | 29.2 (19.4, 41.4) | 26.2 (17.8, 36.8) |
|  | No change | 31.0 (21.2, 42.9) | 33.3 (23.5, 44.9) | 50.8 (39.4, 62.2) | 58.6 (47.1, 69.3) |
|  | Increase | 22.9 (15.1, 33.1) | 20.8 (13.6, 30.4) | 19.9 (12.6, 30.0) | 15.1 (8.6, 25.2) |
| Romania | n | 979 | 997 | 993 | 982 |
|  | Decrease | 47.1 (40.7, 53.5) | 36.8 (30.8, 43.1) | 29.9 (24.3, 36.1) | 40.1 (34.0, 46.6) |
|  | No change | 29.6 (24.1, 35.7) | 38.8 (32.8, 45.1) | 51.4 (45.0, 57.7) | 44.8 (38.5, 51.2) |
|  | Increase | 23.4 (18.4, 29.2) | 24.5 (19.5, 30.3) | 18.7 (14.3, 24.2) | 15.1 (11.0, 20.3) |
| Russia | n | 442 | 452 | 450 | 444 |
|  | Decrease | 41.7 (34.8, 48.8) | 36.4 (29.9, 43.5) | 31.6 (25.4, 38.4) | 35.6 (29.1, 42.8) |
|  | No change | 36.5 (29.9, 43.6) | 46.4 (39.3, 53.6) | 58.2 (51.1, 64.9) | 50.3 (43.1, 57.5) |
|  | Increase | 21.9 (16.0, 29.3) | 17.2 (12.7, 22.8) | 10.3 (7.2, 14.5) | 14.0 (9.0, 21.3) |
| Turkey | n | 191 | 193 | 195 | 193 |
|  | Decrease | 34.8 (23.5, 48.0) | 31.3 (20.6, 44.5) | 25.9 (15.8, 39.4) | 19.8 (10.5, 34.1) |
|  | No change | 42.1 (29.7, 55.6) | 47.6 (35.0, 60.6) | 60.2 (46.9, 72.2) | 68.8 (54.5, 80.3) |
|  | Increase | 23.1 (14.3, 35.2) | 21.1 (12.6, 33.1) | 13.9 (7.1, 25.5) | 11.4 (5.0, 24.0) |
| Ukraine | n | 515 | 528 | 529 | 518 |
|  | Decrease | 34.2 (25.2, 44.6) | 25.3 (17.4, 35.2) | 18.7 (13.0, 26.2) | 25.3 (17.5, 35.1) |
|  | No change | 53.5 (43.2, 63.6) | 61.5 (51.4, 70.8) | 76.6 (68.7, 82.9) | 71.7 (61.9, 79.7) |
|  | Increase | 12.2 (7.5, 19.2) | 13.2 (8.4, 20.1) | 4.7 (2.8, 7.8) | 3.0 (1.6, 5.6) |

Belarus has been excluded as no data was collected on the past-year quantity of alcohol consumed per drink day and the past-year frequency of heavy episodic drinking. HED = heavy episodic drinking.

**Table S5. Key indicators of alcohol use in 17 countries from the Eastern part of the WHO European Region in 2016. Source: WHO Global status report on alcohol and health, 2018 [9].**

|  | Total APC (15+) | Total APC drinker only (15+) | Proportion of unrecorded alcohol in total APC | Decomposition of recorded APC by beverage type | | | | Prevalence of heavy episodic drinking^1^ | Lifetime abstainer | Current drinker^2^ | Former drinker |
| --- | --- | --- | --- | --- | --- | --- | --- | --- | --- | --- | --- |
|  |  |  |  | Beer | Wine | Spirits | Other |  |  |  |  |
| Armenia | 5.5 | 21.3 | 30% | 11% | 7% | 82% | 0% | 10% | 45% | 26% | 29% |
| Bulgaria | 12.7 | 19.2 | 10% | 39% | 17% | 43% | 1% | 33% | 14% | 66% | 20% |
| Belarus | 11.2 | 15.2 | 13% | 23% | 2% | 49% | 26% | 25% | 8% | 74% | 19% |
| Estonia | 11.6 | 15.9 | 10% | 33% | 7% | 50% | 10% | 42% | 12% | 73% | 16% |
| Georgia | 9.8 | 27.9 | 24% | 18% | 43% | 38% | 0% | 17% | 34% | 35% | 31% |
| Croatia | 8.9 | 15.0 | 16% | 44% | 39% | 13% | 3% | 28% | 18% | 60% | 22% |
| Israel | 3.8 | 7.0 | 30% | 54% | 5% | 40% | 1% | 17% | 24% | 55% | 21% |
| Kazakhstan | 7.7 | 19.2 | 25% | 30% | 7% | 63% | 0% | 20% | 32% | 40% | 29% |
| Kyrgyzstan | 6.2 | 24.0 | 34% | 12% | 2% | 86% | 0% | 12% | 46% | 26% | 28% |
| Lithuania | 15.0 | 18.9 | 8% | 44% | 7% | 37% | 12% | 49% | 8% | 79% | 13% |
| Latvia | 12.9 | 17.2 | 15% | 43% | 11% | 40% | 6% | 44% | 10% | 75% | 15% |
| Moldova | 15.2 | 22.8 | 37% | 16% | 57% | 25% | 2% | 27% | 11% | 67% | 22% |
| Montenegro | 8.0 | 14.8 | 21% | 11% | 43% | 44% | 2% | 24% | 23% | 54% | 23% |
| Romania | 12.6 | 18.8 | 17% | 56% | 28% | 16% | 0% | 35% | 13% | 67% | 19% |
| Russia | 11.7 | 20.1 | 31% | 39% | 13% | 39% | 9% | 35% | 27% | 58% | 15% |
| Turkey | 2.0 | 28.5 | 36% | 58% | 9% | 34% | 0% | 1% | 89% | 7% | 4% |
| Ukraine | 8.6 | 13.8 | 36% | 42% | 5% | 52% | 1% | 18% | 13% | 62% | 26% |

Data for Kosovo are not reported by the World Health Organization (WHO). ^1^within the past 30 days. ^2^within the past 12 months.

**Table S6. Key indicators of alcohol use in 17 countries from the Eastern part of the WHO European Region in 2016, organized by sex. Source: WHO Global status report on alcohol and health, 2018 [9].**

|  | Prevalence of current drinker^1^ | | Total APC (15+) | | Total APC drinker only (15+) | | Prevalence of heavy episodic drinking^2^ | |
| --- | --- | --- | --- | --- | --- | --- | --- | --- |
|  | Men | Women | Men | Women | Men | Women | Men | Women |
| Armenia | 38% | 16% | 10.4 | 1.6 | 27.4 | 9.7 | 20% | 3% |
| Bulgaria | 79% | 54% | 21.0 | 4.9 | 26.6 | 9.2 | 52% | 16% |
| Belarus | 79% | 69% | 18.0 | 5.5 | 22.8 | 7.9 | 41% | 12% |
| Estonia | 85% | 63% | 19.4 | 4.9 | 22.9 | 7.9 | 63% | 24% |
| Georgia | 49% | 23% | 17.7 | 2.9 | 36.1 | 12.7 | 29% | 5% |
| Croatia | 74% | 47% | 15.1 | 3.3 | 20.4 | 7.1 | 45% | 12% |
| Israel | 69% | 42% | 6.4 | 1.4 | 9.3 | 3.3 | 29% | 6% |
| Kazakhstan | 54% | 27% | 13.6 | 2.4 | 25.0 | 8.9 | 34% | 7% |
| Kyrgyzstan | 37% | 16% | 11.0 | 1.7 | 29.9 | 10.9 | 20% | 3% |
| Lithuania | 89% | 71% | 24.9 | 6.9 | 27.9 | 9.7 | 71% | 32% |
| Latvia | 87% | 66% | 21.7 | 5.7 | 25.1 | 8.6 | 66% | 26% |
| Moldova | 80% | 55% | 25.2 | 6.1 | 31.7 | 11.1 | 45% | 12% |
| Montenegro | 68% | 40% | 13.5 | 2.8 | 19.8 | 6.9 | 40% | 10% |
| Romania | 80% | 55% | 21.0 | 5.0 | 26.2 | 9.0 | 54% | 17% |
| Russia | 61% | 55% | 18.7 | 5.8 | 30.5 | 10.5 | 48% | 24% |
| Turkey | 11% | 3% | 3.7 | 0.4 | 33.3 | 11.9 | 3% | 0% |
| Ukraine | 69% | 56% | 14.1 | 4.0 | 20.5 | 7.1 | 30% | 7% |

Data for Kosovo are not reported by the World Health Organization (WHO). ^1^within the past 12 months. ^2^within the past 30 days.

s**Table S7. Key indicators of tobacco use in 17 countries from the Eastern part of the WHO European Region in 2018. Source: WHO global report on trends in prevalence of tobacco use 2000-2025, 2019 [10].**

|  | Age-standardised prevalence of tobacco smoking | | | Age-standardised prevalence of cigarette smoking | | |
| --- | --- | --- | --- | --- | --- | --- |
|  | Total | Male | Women | Total | Male | Women |
| Armenia | 26.7% | 51.8% | 1.6% | 25.0% | 48.6% | 1.4% |
| Belarus | 26.6% | 42.8% | 10.4% | 24.8% | 39.9% | 9.7% |
| Bulgaria | 38.9% | 42.5% | 35.3% | 35.8% | 39.5% | 32.1% |
| Croatia | 36.6% | 37.9% | 35.3% | 33.1% | 34.5% | 31.6% |
| Estonia | 30.5% | 36.9% | 24.1% | 27.5% | 33.7% | 21.2% |
| Georgia | 29.7% | 54.2% | 5.2% | 27.0% | 49.3% | 4.8% |
| Israel | 25.5% | 35.2% | 15.8% | 21.7% | 28.9% | 14.5% |
| Kazakhstan | 24.4% | 42.2% | 6.6% | 22.7% | 39.2% | 6.1% |
| Kyrgyzstan | 27.9% | 52.5% | 3.4% | 23.3% | 43.7% | 2.9% |
| Latvia | 36.7% | 49.5% | 24.0% | 32.4% | 43.5% | 21.2% |
| Lithuania | 27.1% | 35.2% | 19.0% | 24.7% | 32.2% | 17.1% |
| Moldova | 25.3% | 44.6% | 6.0% | 22.7% | 40.1% | 5.3% |
| Montenegro | . | . | . | . | . | . |
| Romania | 25.5% | 35.2% | 15.8% | 22.8% | 31.4% | 14.3% |
| Russia | 28.3% | 40.9% | 15.7% | 27.7% | 40.1% | 15.4% |
| Turkey | 29.3% | 41.5% | 17.0% | 27.2% | 38.3% | 16.0% |
| Ukraine | 25.5% | 41.0% | 9.9% | 24.3% | 38.9% | 9.7% |

Data for Kosovo are not reported by the World Health Organization (WHO).

## References

1. Eurostat. Population by educational attainment level, sex and age (1 000) (edat_lfs_9901) [Data set] [Internet]. 2020 [cited 2020 Apr 30]. Available from: https://appsso.eurostat.ec.europa.eu/nui/show.do?dataset=edat_lfs_9901&lang=en

2. Center for Demographic Research Moscow. Russian fertility and mortality database [Data set] [Internet]. 2020 [cited 2020 Jul 20]. Available from: http://demogr.nes.ru/index.php/ru/demogr_indicat/data

3. State Statistics Committee of Ukraine. All-Ukranian Population Census 2001 [Data set] [Internet]. 2001 [cited 2020 Jul 25]. Available from: http://2001.ukrcensus.gov.ua/eng/results/

4. Central Intelligence Agency. The World Factbook 2020 [Data set] [Internet]. 2020 [cited 2020 Jul 20]. Available from: https://www.cia.gov/the-world-factbook/

5. Kosovo Agency of Statistics. 2011 Census. 2011.

6. index mundi. Kosovo Demographics Profile [Internet]. 2020. Available from: https://www.indexmundi.com/kosovo/demographics_profile.html

7. Agency for Strategic planning and reforms of the Republic of Kazakhstan Bureau of National statistics. 2020. Available from: https://stat.gov.kz/

8. National Statistics Office of Georgia. Demographic statistics [Internet]. 2020. Available from: https://www.geostat.ge/en

9. World Health Organization. Global status report on alcohol and health 2018 [Internet]. World Health Organization; 2018 [cited 2019 Mar 29]. Available from: https://www.who.int/substance_abuse/publications/global_alcohol_report/en/

10. World Health Organization. WHO global report on trends in prevalence of tobacco use 2000-2025 [Internet]. Geneva, Switzerland: World Health Organization; 2019 [cited 2021 Nov 23]. Available from: https://apps.who.int/iris/bitstream/handle/10665/330221/9789240000032-eng.pdf

1. All references to Kosovo should be understood within the framework of the UN Security Council Resolution 1244 (1999) [↑](#footnote-ref-1)
2. All references to Kosovo should be understood within the framework of the UN Security Council Resolution 1244 (1999) [↑](#footnote-ref-2)
3. All references to Kosovo should be understood within the framework of the UN Security Council Resolution 1244 (1999) [↑](#footnote-ref-3)
